# LSM14B controls oocyte mRNA storage and stability to ensure female fertility

**DOI:** 10.1007/s00018-023-04898-2

**Published:** 2023-08-14

**Authors:** Li-Ying Shan, Yu Tian, Wen-Xiang Liu, Hai-Tao Fan, Feng-Guo Li, Wen-Juan Liu, Ang Li, Wei Shen, Qing-Yuan Sun, Yong-Bin Liu, Yang Zhou, Teng Zhang

**Affiliations:** 1 State Key Laboratory of Reproductive Regulation and Breeding of Grassland Livestock (R2BGL), College of Life Sciences, Inner Mongolia University, Hohhot, 010070 China; 2 Fertility Preservation Lab, Guangdong-Hong Kong Metabolism & Reproduction Joint Laboratory, Reproductive Medicine Center, Guangdong Second Provincial General Hospital, Guangzhou, 510317 China; 3 College of Life Sciences, Institute of Reproductive Sciences, Qingdao Agricultural University, Qingdao, 266109 China

**Keywords:** *Lsm14b*, P-body-like granules, mRNA stability, Primordial follicle assembly, Oocytes

## Abstract

Controlled mRNA storage and stability is essential for oocyte meiosis and early embryonic development. However, how to regulate mRNA storage and stability in mammalian oogenesis remains elusive. Here we showed that LSM14B, a component of membraneless compartments including P-body-like granules and mitochondria-associated ribonucleoprotein domain (MARDO) in germ cell, is indispensable for female fertility. To reveal loss of LSM14B disrupted primordial follicle assembly and caused mRNA reduction in non-growing oocytes, which was concomitant with the impaired assembly of P-body-like granules. 10× Genomics single-cell RNA-sequencing and immunostaining were performed. Meanwhile, we conducted RNA-seq analysis of GV-stage oocytes and found that *Lsm14b* deficiency not only impaired the maternal mRNA accumulation but also disrupted the translation in fully grown oocytes, which was closely associated with dissolution of MARDO components. Moreover, *Lsm14b*-deficient oocytes reassembled a pronucleus containing decondensed chromatin after extrusion of the first polar body, through compromising the activation of maturation promoting factor, while the defects were restored via WEE1/2 inhibitor. Together, our findings reveal that *Lsm14b* plays a pivotal role in mammalian oogenesis by specifically controlling of oocyte mRNA storage and stability.

**Supplementary Information:**

The online version contains supplementary material available at 10.1007/s00018-023-04898-2.

## Introduction

In mammals, the number of available primordial follicle is a major factor which determines the reproductive life span. The number of available oocytes is determined immediately after birth in mice. Therefore, the establishment of primordial follicle pool is important for reproductive sustainability [1]. The primordial follicle populations are considered a finite resource, with any disturbances in the assembly process potentially affecting a woman’s overall oocyte availability and increasing the risk of conditions such as premature ovarian insufficiency [2, 3]. It is noteworthy that the oocytes stored in the primordial follicles, referred to as non-growing oocytes (NGO), while only a limited number of oocytes are activated and developed, referred to as growing oocytes (GO) [4]. Along with the oocyte growth, it accumulates maternal mRNA in the cytoplasm before reaching fully grown oocytes (FGO) [5].

Maternal mRNA required for oocyte maturation and early embryonic development must accumulate continuously and steadily during oocyte growth [4, 6, 7]. However, the mechanism of maternal mRNA storage remains elusive during oogenesis. In general, maternal mRNA associates with numerous ribonucleoprotein complexes and accumulates in membraneless compartments by phase separation, such as P-body-like granules and mitochondria-associated ribonucleoprotein domain (MARDO) [8–12]. Previous studies have established the presence of scattered P-body-like granules in the oocyte cytoplasm, which serves as a repository for genes associated with the primordial follicle during the establishment of the primordial follicle pool [13]. Constituting processing bodies (P-bodies) are a major class of proteins (LSM1-7, DCP1/2, EDC3, EDC4, UPF1, YTHDF2, DDX6, LSM14A, YBX2) associated with mRNA demethylation [14–18] and another class including RNA-binding proteins (4E-T/EIF4ENIF1, CPEB1, DYNC1H1) [19–21] that promote translation inhibition [22, 23]. A recent study has demonstrated that the absence of DDX6, the central component of P-body-like granules, impairs the primordial follicle assembly, indicating that P-body-like granules is essential in maintaining primordial follicle assembly [13]. Notably, LSM14B interacts specifically with DDX6, which is detected in polysomes and stagnant translation-initiating complexes, playing a role in inhibiting translation [24]. Subsequently, together with ZAR1, YBX2, and 4E-T as core components of MARDO, they were reported to store mRNA in mammalian FGO, ensuring stable maturation of oocytes. *Zar1* is one of the maternal-effect genes that regulate transcription activation and precisely control early oogenesis and maternal mRNA homeostasis [6, 25]. Similarly, *Ybx2* acts as oocyte-specific gene that regulates primordial follicle assembly and follicle growth [26, 27]. Given that the accumulation of maternal mRNA begins at an early oogenesis [4], it is reasonable to infer that these membraneless compartment core protein involved in maternal mRNA storage already play a role in the early stage of oogenesis [28–30].

LSM14B, represented by RAP55, belongs to the RNA-associated protein and is involved in the assembly of P-body and MARDO [24, 31]. Previous results have found that LSM14B affects meiotic processes and oocyte maturation by regulating the activities of spindle assembly checkpoints and maturation promoter factor (MPF) [32]. However, there is currently a lack of direct evidence to support the contribution of LSM14B to the storage of maternal mRNA during oogenesis.

In this study, we found that LSM14B is one of the components of P-body-like granules and forms aggregate-like foci that co-localized with DDX6 during the primordial follicle formation. Depletion of LSM14B leads to P-body-like granules structure destruction and mRNA decay and affects primordial follicle assembly and maternal mRNA accumulation. Moreover, we found that reduced MPF activity in *Lsm14b* knockout (KO) oocytes led to the formation of pronucleus (PN) after extrusion of the first polar body (PB1), which was eliminated by activation of CDK1. Our results confirm that LSM14B is specifically expressed in oocytes and is involved in the stabilization and storage of maternal mRNA.

## Materials and methods

### Animals

All mouse strains had a C57BL/6 background. *Lsm14b* KO mouse strains were made using the CRISPR/Cas9 system, as shown in Fig S2B. Mice were maintained under specific pathogen-free conditions in a controlled environment at 22 ± 2℃ with a 12/12 h light/dark cycle, 45–60% humidity, and free access to food and water. Mice were genotyped by PCR using primers as shown in Table S1, which produces a 453 and 600 bp PCR product for the wild type (WT) and *Lsm14b* KO alleles, respectively. All animal experiments were approved and performed in line with the Animal Care and Use Committee at Inner Mongolia University (IMU-MOUSE-2023-013).

### Co-immunoprecipitation

The ovarian samples were lysed by adding 50 mg of tissue in 600 μl IP buffer containing 20 mM Tris–HCl, 10 mM EDTA, 1 mM EGTA, 150 mM NaCl, 0.05% Triton X-100, 0.05% NP-40, and 1 mM PMSF. Prior to use, protease (Sigma-Aldrich, P8340, 1:100) and phosphatase inhibitors (Sigma-Aldrich, P5726, 1:500) were added. Protein A&G agarose beads were washed and then incubated with anti-IgG and anti-LSM14B at 4 °C for 4 h, respectively. Ten microliters of both IgG and LSM14B rabbit antibodies were used. Antibody combined beads were collected and incubated with lysates at 4 °C overnight. Beads were washed three times and boiled for 5 min. The SDS-PAGE was run for immune blotting, followed by matrix-assisted laser desorption/ionization time of flight mass spectrometry (MALDI/MS) analysis. The gel samples were sent to the proteomics facility at Shanghai Applied Protein Technology to undergo MALDI/MS. Original blots can be found in the Source data file.

### Immunohistochemistry

Ovarian samples were collected from each group, fixed with 4% paraformaldehyde, embedded in paraffin, and continuously sectioned at a thickness of 5 μm. Tissue sections were subjected to gradient alcohol dewaxing and incubated with blocking solution (3% BSA, 10% normal donkey serum in TBST) for 30 min. After overnight incubation with primary antibodies (DDX4, LSM14B, and DDX6 in Table S2) at 4 °C followed by incubation with fluorescein-conjugated secondary antibody (Table S2), nuclei were stained with DAPI after washing with TBST.

### Hematoxylin–eosin (H&E) staining

Ovaries were fixed for overnight at 4℃ temperature in Bouin’s fixative (Sigma, HT10132), followed by ethanol dehydration, xylene clarification, and paraffin embedding. Ovarian blocks were serially sectioned at 5 μm thickness. After drying at 37 °C, the samples were stained with Lillie-Mayer hematoxylin (Solarbio, G1080, China) and Eosin (Zsbio, ZLI-9613, China). Follicles at different stages of development were counted in every three sections of the whole ovary, and the total number of each stage follicles was calculated as described previously. To avoid double counting the same follicle, only follicles containing oocytes with visible nuclei were counted and classify the follicles according to their characteristics.

### Fertility identification

Fertility tests were carried out by mating 8-week-old WT and *Lsm14b* KO female mice with WT male mice for 6 months. The number of pups for each cage was recorded at birth, and the average number of pups per cage was calculated.

### Tissue immunofluorescence

After fixing the freshly isolated ovaries in 4% paraformaldehyde (PFA) prepared in PBS overnight then infiltrated in paraffin (Leica, 39,601,095, Germany), sample sections of 5 μm in thickness were blocked at room temperature for 30 min after gradient rehydration and antigen retrieval and incubated with primary antibodies (LSM14B, DDX4 in Table S2) overnight at 4˚C. After 3 washes in TBST, the sections were incubated with secondary antibodies of goat anti-rabbit IgG (Table S2) for 30 min at room temperature. Expressed immunoreactive signals were visualized by DAB kit (ZSGB-BIO, ZLI-9017, China) and nuclei were counterstained with hematoxylin. After dehydration, the slide was immediately sealed with neutral balsam.

### Single-cell libraries and sequencing

With qualified single-cell sample, cells in 0.04% BSA in PBS were loaded onto 10 × Chromium chip, and single-cell gel beads in emulsion were generated by using Single Cell 30 Library and Gel Bead Kit V2 (10 × Genomics Inc., 120,237, Pleasanton, California, USA). Following the manufacturer’s instructions, single-cell RNA-seq libraries were constructed, and pair-end 150 bp sequencing was performed to produce high-quantity data on an Illumina HiSeq X Ten (Illumina, San Diego, California, USA).

The raw data were analyzed and aggregated using Cell Ranger (version 4.0) to obtain barcode tables and gene expression matrices. Afterward, we imported the matrix into Seurat (version 4.0.2) for quality control. Data integration was done through the “FindIntegrationAnchors” and “IntegrateData” functions. To divide the various types of cell clusters, the “RunUMAP” function was used for cell clustering and visualization. In addition, differential analysis of germ cells and granulosa cells in the WT and *Lsm14b* KO ovaries was done by “FindMakers.” We considered genes with *P* < 0.05 and |Log_2_(FC)|> 0.25 to be significantly different. Next, we performed pseudotime cell trajectory construction on the gene count matrix based on the R package Monocle (version 2.18.0). This process used the “reduceDimension” and “orderCells” functions.

### Superovulation and fertilization

In superovulation, female mice (6 to 8 weeks old) were intraperitoneally injected with 5 IU of PMSG for 48 h. Subsequently, they were then injected with 5 IU of hCG, and oocyte cumulus complexes were removed from the oviducts 15 h later, and the numbers of oocytes were counted after digestion with 0.3% hyaluronidase for 10 min. For in vitro fertilization (IVF), TYH medium (EasyCheck, M2050, China), HTF medium (EasyCheck, M1130, China), and in vitro maturation (IVM) medium (EasyCheck, M2115, China) were put into 37 °C in 5% CO_2_ atmosphere overnight. On the second day, the epididymis of adult male mice was removed and transferred to the capacitation fluid for 1 h. Subsequently, the oocytes were transferred into the fertilization drop, and the spermatozoa in 10 μl TYH medium were taken out and added to the fertilization drop. After the fertilization for 6 h, the unfertilized sperm were washed with IVM medium, and the fertilized oocytes were transferred into the HTF medium. Formation of 2-cell stage embryos was scored 24 h after IVF and development of 2-cell embryos to the blastocyst stage was scored 3–5 days after culture.

### Oocyte culture

The 6-week-old female mice were injected with 5 IU of PMSG and humanely euthanized 48 h later. The GV-stage oocytes were harvested in M2 medium (Sigma, M7167, Japan) and cultured in 100 μl of M2 medium covered with mineral oil (Sigma, M8410, Japan) at 37 °C in a 5% CO_2_ atmosphere. During culture, the stage of GV, Pro-metaphase I (pre-MI), Metaphase I (MI), Anaphase I (AI), Telophase I (TI), and Metaphase II (MII) oocytes were collected at the time points of 0, 4, 6, 8, 10, and 14 h.

### Confocal microscopy of mouse oocytes

Oocytes were fixed with 4% paraformaldehyde. Then they were permeabilized with 0.3% Triton X-100 in PBS for 30 min. After being blocked with 1% bovine serum albumin in PBS, the oocytes were incubated with primary antibodies for 1 h and sequentially incubated with anti-α-tubulin-FITC antibody (Sigma, F2168, Japan) overnight. Finally, the oocytes were washed three times with the TBST and then stained with Hoechst 33,342 (Solarbio, C0031, China) for 15 min. Imaging was carried out using a confocal laser-scanning microscope (A1R, Nikon, Japan).

### Western blot analysis

Proteins were collected from P3 ovaries and GV and MII oocytes by adding RIPA (addition of protease inhibitors) and vortexing on ice, followed by boiling with an equal volume of 2 × SDS loading buffer for 10 min. Samples were separated by SDS-PAGE, transferred to PVDF membranes, blocked with 5% non-fat dry milk in TBST for 1 h at room temperature, and incubated with primary antibody (LSM14B, DDX6, p-CDK1(Y15), CCNB1, DDX4, β-ACTIN in Table S2) at 4 °C overnight. Then, membranes were washed with TBST and incubated with secondary antibodies for 1 h at room temperature. Chemiluminescence was performed using a BeyoECL Plus Kit (Beyotime, A0018), as directed by the manufacturer.

### RNA extraction and real-time quantitative PCR (RT-qPCR)

Total RNA was extracted from oocytes, testis, muscle, brain, kidney, lung, spleen, and liver of WT and *Lsm14b* KO mice according to the instructions of the EasyPure® RNA Kit (TransGen Biotech, ER101, Beijing, China), and the subsequent cDNA samples were subjected to reverse transcription Kit (TransGen Biotech, AT311) and RT-qPCR was performed in 10 µl reactions using a Light Cycler 480II System (Roche Diagnostics). The 2^(−ΔΔCt)^ method was used to analyze RT-qPCR data and data were normalized to *Gapdh.* Primer sequences for the targeted mouse genes are listed in Table S1.

### RNA-seq analysis

Transcriptomic analysis of WT and *Lsm14b* KO GV-stage oocytes was performed according to the protocol provided by the company. Briefly, per 20 fully grown oocytes were removed into individual tubes that contained 10 µl of lysis buffer. According to the manufacturer’s instructions, reverse transcription reaction was carried out directly on the whole cell lysate using Illumina Sequencing-HV kits (Takara Bio Inc, Japan). Sequencing libraries were constructed from amplified cDNA. Barcoded libraries were pooled and sequenced on Illumina HiSeq 4000 platform with 150 bp pair-end reads. All the raw reads were trimmed to remove low-quality bases and adaptor sequences. The clean reads were then mapped to the mouse genome with STAR (version 2.7.10). The expression levels of each gene were quantified using featureCounts software (version 2.0.3). Differentially expressed genes were calculated using default parameter of DESeq2 (version 1.30.1).

### Proteomic analysis

For each group of 500 GV-stage oocytes, SDT lysis method was used to extract protein, and then BCA method was used for protein quantification. An appropriate amount of protein was taken from each sample for trypsin digestion by filter-aided proteome preparation (FASP) method, using C18 Cartridge. The peptides were desalted, lyophilized, and reconstituted with 40 μl of 0.1% formic acid solution, and the peptides were quantified (OD280). Each sample was separated using the NanoElute HPLC liquid system at nanoliter flow rates. The samples were chromatographically separated and analyzed by mass spectrometry using a timsTOF Pro mass spectrometer. The detection method was positive ion, the ion source voltage was set to 1.5 kV, and both MS and MSMS were detected and analyzed by TOF. The MS scan range was set to 100–1700 m/z. The data acquisition mode adopts the parallel accumulation serial fragmentation (PASEF) mode. After the first-order mass spectrum was acquired, 10 ions were collected in the PASEF mode, with a cycle window time of 1.17 s. The raw data of mass spectrometry analysis were RAW files, and MaxQuant software (version 1.6.14) was used for library identification and quantitative analysis. Differentially expressed proteins were calculated using default parameter of DESeq2 (version 1.30.1).

### Gene Ontology and KEGG enrichment analysis

The R package clusterProfiler (version 3.18.1) was used to perform Gene Ontology and KEGG enrichment analysis on the differential genes [33], and the threshold was set to “*P* < 0.05” to match the highly correlated biological processes and signaling pathways of the differential genes.

### Statistical analysis

All statistical analyses were performed using GraphPad Prism software (version 8.0). Data are presented as means ± SEM. Student’s t-*t*ests were used to compare differences between two groups, with *P* < 0.05 defined as significantly different. One-way analysis of variance (ANOVA) followed by Tukey’s multiple comparison’s test was used to assess the statistical significance of differences among three groups. Differential gene/protein were defined as those with variable importance in the projection value greater than 1.0 and *P*-adjusted < 0.05.

## Results

### *LSM14B *is a component of *P*-body-like granules and MARDO within germ cells

**Fig. 1:**
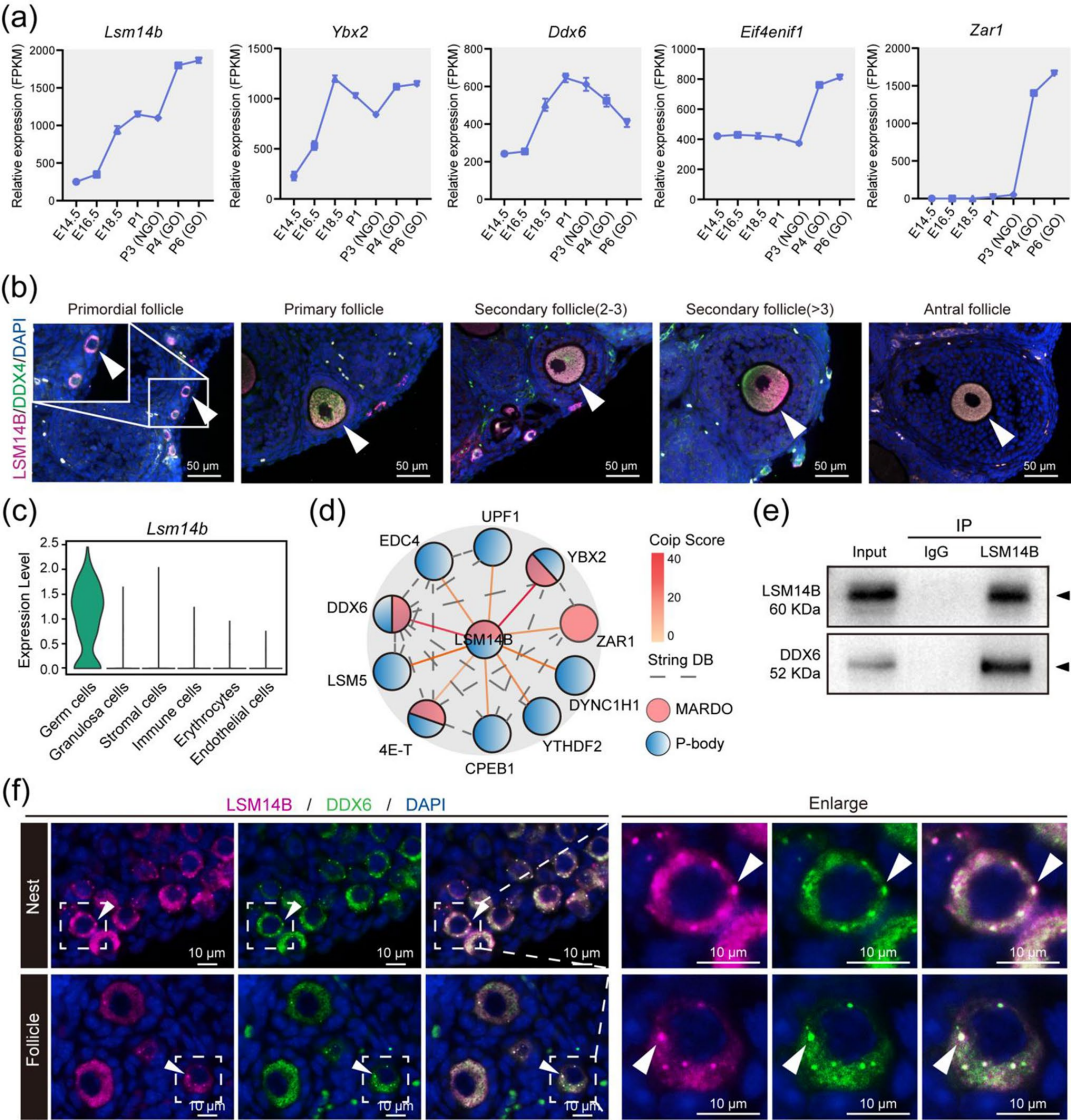
**LSM14B exhibits oocyte-specific expression and as an integral constituent of both P-body-like granules and MARDO. a** Gene expression dynamics of MARDO in oocyte at embryonic day 14.5 (E14.5), E16.5, E18.5, postnatal day 1 (P1), P3, P4, and P6. The averaged FPKM values of each of the indicated genes are shown. **b** Immunostaining of LSM14B (magenta) and germ cell specific markers DDX4 (green) in different classes of follicles. Cell nuclei were counterstained with 4’,6-diamidino-2-phenylindole (DAPI, blue). Scale bar, 50 μm. **c** Violin diagram showed that *Lsm14b* was specifically expressed in ovarian germ cells. **d** Co-IP followed by mass spectrometry analysis was carried to identify the interactions between LSM14B and protein associated with the membraneless compartment, which contains pink-indicated MARDO and blue-indicated P-body. **e** Co-IP followed by Western blot was performed to prove the interaction between LSM14B and DDX6. **f** Representative image of immunostaining of oocytes in nest and follicle with LSM14B (magenta) and DDX6 (green) in P3 ovaries. Nuclei were counterstained with DAPI (blue). Scale bar, 10 μm

MARDO have been shown to regulate maternal mRNA storage, translation, and decay in fully grown oocytes (FGOs) [34]. Notably, we found that gene expression levels of *Lsm14b*, *Ybx2*, *Ddx6*, *Eif4enif1*, and *Zar1*, which are the compositions of MARDO, were significantly increased in oocytes from E14.5 to postnatal day 6 (P6) (Fig. 1a) [4], indicating that MARDO members may be functional in early oogenesis. Immunostaining analysis demonstrated that LSM14B co-localized with germ cell-specific marker DDX4 in all stages of follicles, which aligns with the findings of transcriptomic analysis of ovarian cells (Fig. 1b), suggesting a potential role for LSM14B in oocyte. Furthermore, to identify the potential interacting partners of LSM14B, we performed co-immunoprecipitation (Co-IP) of LSM14B protein followed by mass spectrometry (MS) analysis and revealed that the interacting proteins, including DDX6, YBX2, CPEB1, ZAR1, EDC4, LSM5, and 4E-T, are not only involved in the constitution of MARDO but also are the part of P-body (Fig. 1d, Supplementary Fig. 1a, b). Previous studies have established that RNA-binding proteins YBX2 [35] and ZAR1 [34] are required for maternal mRNA storage and metabolism. The DDX6 and 4E-T interaction mediates translational repression and de novo P-body assembly [19]. Previous study also indicated that LSM14B specifically interacts with DDX6, which plays a pivotal role in the regulation of mRNA metabolism during the early developmental stage of *Xenopus* oocyte [24]. Moreover, loss of DDX6 disturbs the P-body-like granules formation and severely impairs the assembly of primordial follicle in mice [13]. Similarly, the Co-IP experiment indicated that LSM14B indeed interacts with DDX6 in mouse oocytes (Fig. 1e). Importantly, immunofluorescence staining showed that LSM14B was co-localized with DDX6 and presented aggregate-like foci in germ cells (Fig. 1f). These observations suggest that LSM14B is involved in the composition of the membraneless compartment including P-body-like granules and MARDO, although the role of LSM14B remained unknown in mammalian oocytes.

### Knockout of *Lsm14b* compromises female fertility and primordial follicle pool

**Fig. 2:**
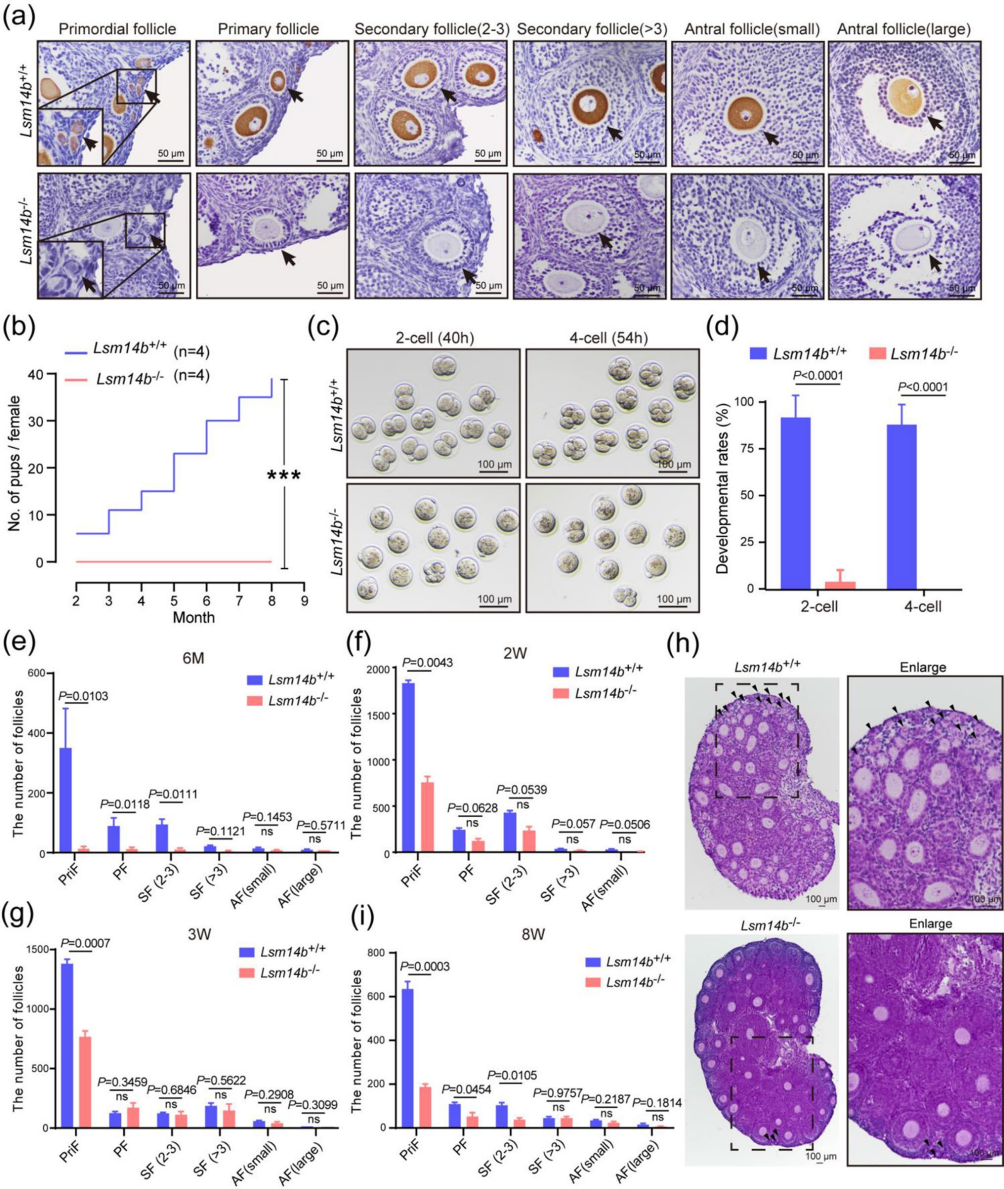
**Knockout of** *Lsm14b* **compromises female fertility concomitant with a decline in primordial follicle reserve. a** Representative immunohistochemistry of LSM14B protein (dark brown) of different classes of follicles in the WT and *Lsm14b* KO mice. **b** Cumulative numbers of pups per female showing fertility of WT and *Lsm14B* KO female mice. Data are the mean ± SEM (*n* = 4 females for each genotype). **c** Representative images of 2-cell embryos and 4-cell embryos formed by ovulated oocytes after in vitro fertilization of WT mice and *Lsm14b* KO mice. **d** The proportion of 2-cell embryos and 4-cell embryos in WT mice and *Lsm14b* KO mice. Data are the mean ± SEM (*n* = 3 biologically independent repeats). **e** Quantification of the number of different classes of follicles (primordial follicle: PriF; primary follicle: PF; secondary follicle: SF; antral follicle: AF) in ovaries of 6 months WT mice and *Lsm14b* KO mice. Data are the mean ± SEM (*n* = 3 and 5 females for WT and *Lsm14b* KO for 6 months mice, respectively). ns: non-significant. **f–g** Quantification of the number of different classes of follicles in ovaries of WT mice and *Lsm14b* KO mice at 2 and 3 weeks, respectively. Data are the mean ± SEM (*n* = 2 females for each genotype for 2 weeks mice, *n* = 3 females for each genotype for 3 weeks mice). ns: non-significant. **h** Representative H&E image of different classes of follicles in 3 weeks ovaries. The arrows represent primordial follicle. Scale bar, 100 μm. **i** Quantification of the number of different classes of follicles in ovaries of 8 weeks WT mice and *Lsm14b* KO mice. Data are the mean ± SEM (*n* = 3 females for each genotype for 8 weeks mice). ns: non-significant

The expression level of *Lsm14b* in different tissues was detected by RT-qPCR and found to be highly expressed in oocyte (Supplementary Fig. 2a). To examine the functions of LSM14B in oocyte, we generated *Lsm14b* knockout (*Lsm14b* KO) mice (Supplementary Fig. 2b–d). Predictably, LSM14B protein expression was not detected in different classes of follicles in mutant mice (Fig. 2a). We found no significant changes in body weight, ovarian size, or ovulation number in 8-week-old female *Lsm14b* KO mice (Supplementary Fig. 2e–g). Fertility testing revealed that *Lsm14b* KO female mice were completely infertile after crossing with WT male mice (Fig. 2b). Eggs ovulated by 8-week-old *Lsm14b* KO mice could be fertilized. Nevertheless, over 90% of the *Lsm14b*^♀−/♂+^ embryos failed to develop to the 2-cell stage, compared to the WT group (Fig. 2c, d). Notably, we also found that a number of follicles in the ovaries of *Lsm14b* KO mice at 6 months were nearly exhausted compared to WT mice (Fig. 2e; Supplementary Fig. 2 h), suggesting that *Lsm14b* KO caused premature ovarian failure. Given that the LSM14B presented aggregate-like foci in the primordial follicles, we detected the numbers of the primordial follicle. The results show that the numbers of primordial follicle in the ovaries of *Lsm14b* KO mice at 2, 3, and 8 weeks were significantly reduced compared to WT mice (Fig. 2f–i). Based on the results of primordial follicle loss in mutant ovaries, we concluded that *Lsm14b* plays an integral role during the primordial follicle assembly.

### *LSM14B* is essential for primordial follicle assembly

**Fig. 3:**
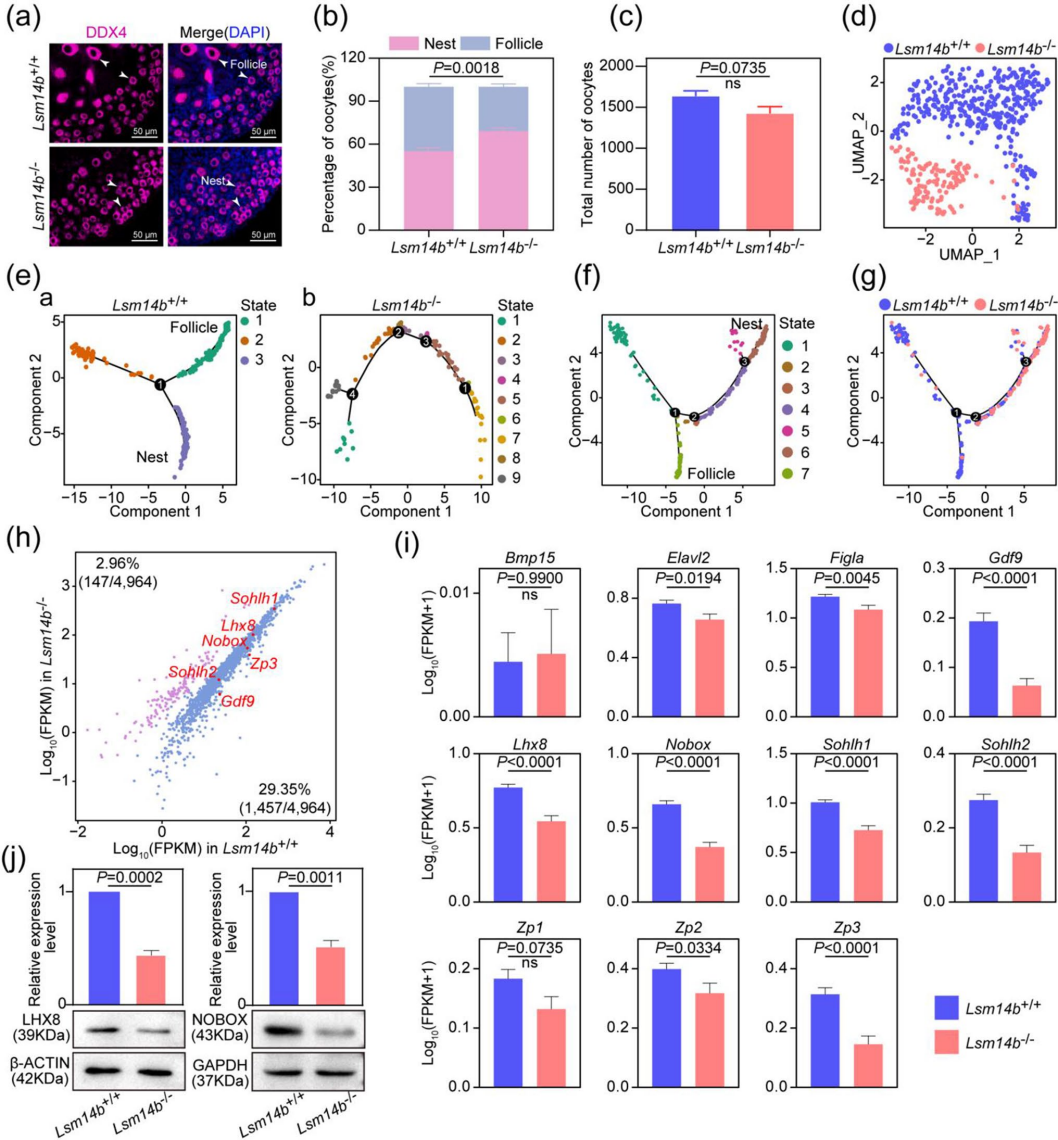
**Loss of LSM14B impaired the assembly of primordial follicle assembly. a** Representative image of nests and follicles staining of ovaries sections from P3 mice. Germ cells are labeled with MVH (magenta), and nucleus was counterstained with DAPI (blue). Scale bar, 50 μm. Data are the mean ± SEM (*n* = 5 for each genotype). **b** The percentage of germ cells within nests and follicles in WT and *Lsm14b* KO ovaries. **c** The count of germ cells in WT and *Lsm14b* KO ovaries. Data are the mean ± SEM (*n* = 3 for each genotype). ns: non-significant. **d** Clustering of germ cell population with UMAP, colored based on sample groups. **e** (a) Single-cell pseudotime developmental trajectory of WT germ cells, (b) Single-cell pseudotime developmental trajectory in *Lsm14b* KO germ cells, which are colored according to cell development state. **f** The pseudotime trajectory of germ cell populations colored by seven states. **g** The pseudotime trajectory of germ cell population colored by two sample groups. **h** The scatter plot shows how genes whose expression levels increased more than threefold from E16.5 to P3 differ between WT and *Lsm14b* KO P3 oocytes. **i** Relative expression levels of genes involved in primordial follicle assembly in ovaries. **j** Detection of LHX8, NOBOX and GAPDH proteins of P3 ovaries by Western blot. GAPDH (for LHX8 and NOBOX control expression)

Primordial follicle assembly is a finely regulated complex process, and it determines the length of the reproductive life in female. We observed that the percentage of germ cells in primordial follicle was dramatically decreased, and a total number of germ cells were not lost in the P3 ovaries of *Lsm14b* KO mice (Fig. 3a–c). These results indicated that primordial follicle assembly is severely compromised in *Lsm14b* KO ovaries.

To characterize the temporal and mechanistic features of impaired primordial follicle assembly in *Lsm14b* KO ovaries, a 10 × Genomics single-cell atlas of ovary was performed, and then we identified and extracted germ cell clusters based on data quality control, UMAP cluster analysis, and known cell-specific markers [1] (Supplementary Fig. 3a–d). Notably, germ cell clusters were labeled as nine cell clusters by UMAP analysis, whereas germ cells in WT and *Lsm14b* KO mice were distributed in distinct cell clusters (Fig. 3d, Supplementary Fig. 3e). This suggested that the absence of LSM14B induced divergent transcriptional profiling of germ cells.

To explain this abnormal cellular distribution, we performed a pseudotime trajectories analysis. There are three states of cells at P3 in the germ cells of WT mice. Based on the dynamic expression of *Id1* and *Ooep* on pseudotime trajectories, state 1 was assigned as “Follicle” and state 3 as “Nest” (Fig. 3e (a), Supplementary Fig. 3f (a)). By contrast, germ cells of *Lsm14b* KO mice adopted different branches and had a multipath differentiation process (Fig. 3e (b); Supplementary Fig. 3f (b)). It is worth noting that when the germ cells of the two groups were placed on the same pseudotime developmental process, it could be found that most of the *Lsm14b* KO germ cells were arrested at the “Nest” stage and could not become “Follicle” (Fig. 3f-g; Supplementary Fig. 3 g). Interestingly, granulosa cells, an important player in primordial follicle assembly, were also affected by deletion of *Lsm14b*. Two groups of granulosa cells were distributed in different cell clusters (Supplementary Fig. 4a, b). And granulosa cells in WT P3 ovaries arranged in a linear trajectory, while the development process of *Lsm14b* KO granulosa cells was disrupted (Supplementary Fig. 4c). These results further indicated that transcriptional profiling of primordial follicle assembly was disrupted in *Lsm14b* KO mice.

To evaluate the influence of *Lsm14b* deficiency on gene expression, a time-course transcriptomic differences were assessed by scRNA-seq analyses in WT oocytes from E16.5, P0, and P3. We found that consistent with the timing of primordial follicle formation, 4,964 genes (more than threefold) were greatly increased in germ cell from E16.5 to P3. Among these 4,964 genes, there were tenfold more genes with significantly down-regulated expression levels than with significantly up-regulated expression levels in *Lsm14b* KO P3 oocytes compared with WT P3 oocytes (Fig. 3h). We observed that the expression levels of genes associated with the assembly and quality of primordial follicle in the *Lsm14b* KO ovaries were significantly lower than in the WT ovaries (Fig. 3i). Meanwhile, WB results showed that the protein levels of LHX8 and NOBOX in 3d *Lsm14b* KO ovaries were also significantly decreased (Fig. 3j). Our results confirm that LSM14B is indispensable for the primordial follicle assembly process.

### *Lsm14b* deletion impaired the assembly of *P*-body-like granules

**Fig. 4:**
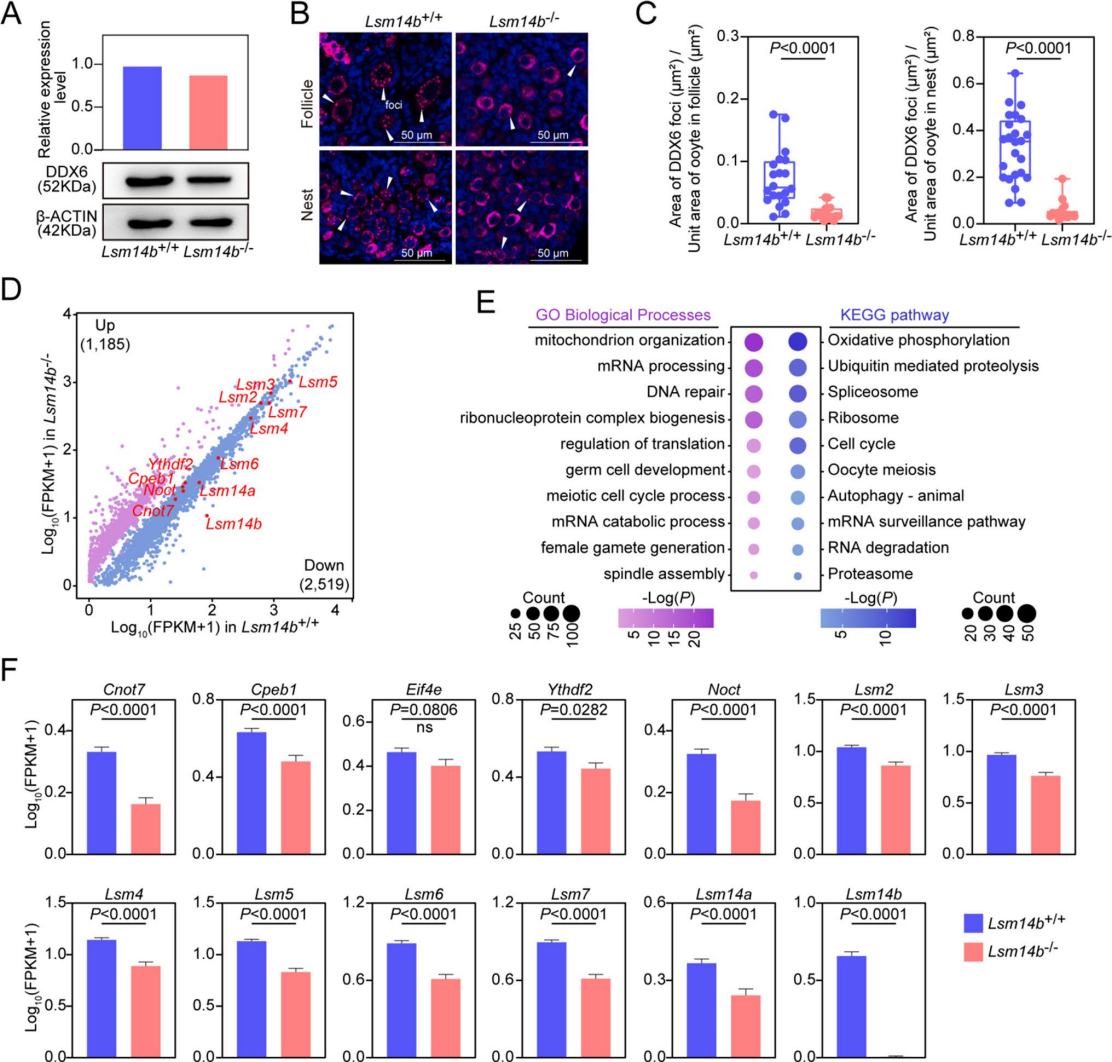
**Disrupted assembly of P-body-like granules in** *Lsm14b* **KO oocytes. a** Detection of DDX6 and β-ACTIN proteins expression level of P3 ovaries by Western blot. **b** Immunostaining of DDX6 (magenta) in nest and follicle of P3 ovaries. The arrows represent the foci of DDX6. Cell nuclei were counter stained with DAPI (blue). Scale bar, 50 μm. **c** Quantification of nest and follicle containing DDX6 foci. Data are the mean ± SEM (*n* = 4 for each genotype). **d** Scatter plot comparing transcripts between WT and *Lsm14b* KO from P3 ovaries. Transcripts decreased or increased in the *Lsm14b* KO sample, highlighted in blue or purple, respectively. **e** Gene Ontology and KEGG enrichment analysis of down-regulated genes list. **f** Relative expression levels of P-body-like granules related genes in P3 ovaries

It was previously reported that P-body-like granules are critical for primordial follicle assembly in mice [13]. Given that LSM14B was co-localized with DDX6 and formed aggregate-like foci in newborn ovaries, we investigated whether the absence of LSM14B affects the assembly of P-body-like granules. Western blot analyses showed that DDX6 was present at normal levels in *Lsm14b* KO group compared with the WT group (Fig. 4a). Notably, immunostaining of P-body-like granules components revealed that the assembly of P-body-like granules was impaired in *Lsm14b* KO ovaries (Fig. 4b). Next, we measured the area of DDX6 foci per unit area (μm^2^) of nest and follicle. Statistical analysis revealed a significant reduction in DDX6 lesion area in *Lsm14b* KO ovaries relative to WT ovaries (Fig. 4c). The results suggest that *Lsm14b* may be involved in the assembly of P-body-like granules in oocytes.

Considering the P-body components play crucial roles in mRNA metabolism [18, 30], we investigated whether disruption of P-body-like granules affects dynamics of mRNA in oocytes. The scRNA-seq data revealed that more transcripts were found to be down-regulated by at least 50% (2519 down and 1185 up, respectively) in *Lsm14b* KO NGO, as compared with the WT NGO (Fig. 4d). Gene enrichment analysis revealed that the down-regulated mRNAs were mainly associated with processes related to “mRNA metabolism and translation,” “oocyte meiosis and cell cycle,” and “mitochondrion organization and oxidative phosphorylation” (Fig. 4e). It is worth noting that P-bodies were highlighted to be involved in processes similar to “mRNA metabolism and translation” and “oocyte meiosis and cell cycle” [22, 36, 37]. Interestingly, similar dynamics of mRNA in granulosa cells were also exhibited in *Lsm14b* KO group (Supplementary Fig. 4d–f). Moreover, we found the decreased mRNA expression levels of several of P-body-like granules components in *Lsm14b* KO oocytes (Fig. 4f). These results suggest that many mRNAs involved in the regulation of oocyte development are stored in P-body-like granules and that the absence of LSM14B not only disrupts P-body-like granules function but also causes mRNA reduction.

### Loss of *LSM14B* disrupts maternal mRNA accumulation and translation in FGO

Previously results indicated that deletion of ZAR1 impaired maternal mRNA accumulation in oocyte, and loss of ZAR1 disrupts MARDO formation and mRNA storage, translation, and degradation during oocyte maturation [6]. Since LSM14B is predicted RNA-binding proteins [38], and LSM14B deletion causes mRNA reduction, we investigated the effects of LSM14B deletion on maternal mRNA in FGO.

**Fig. 5:**
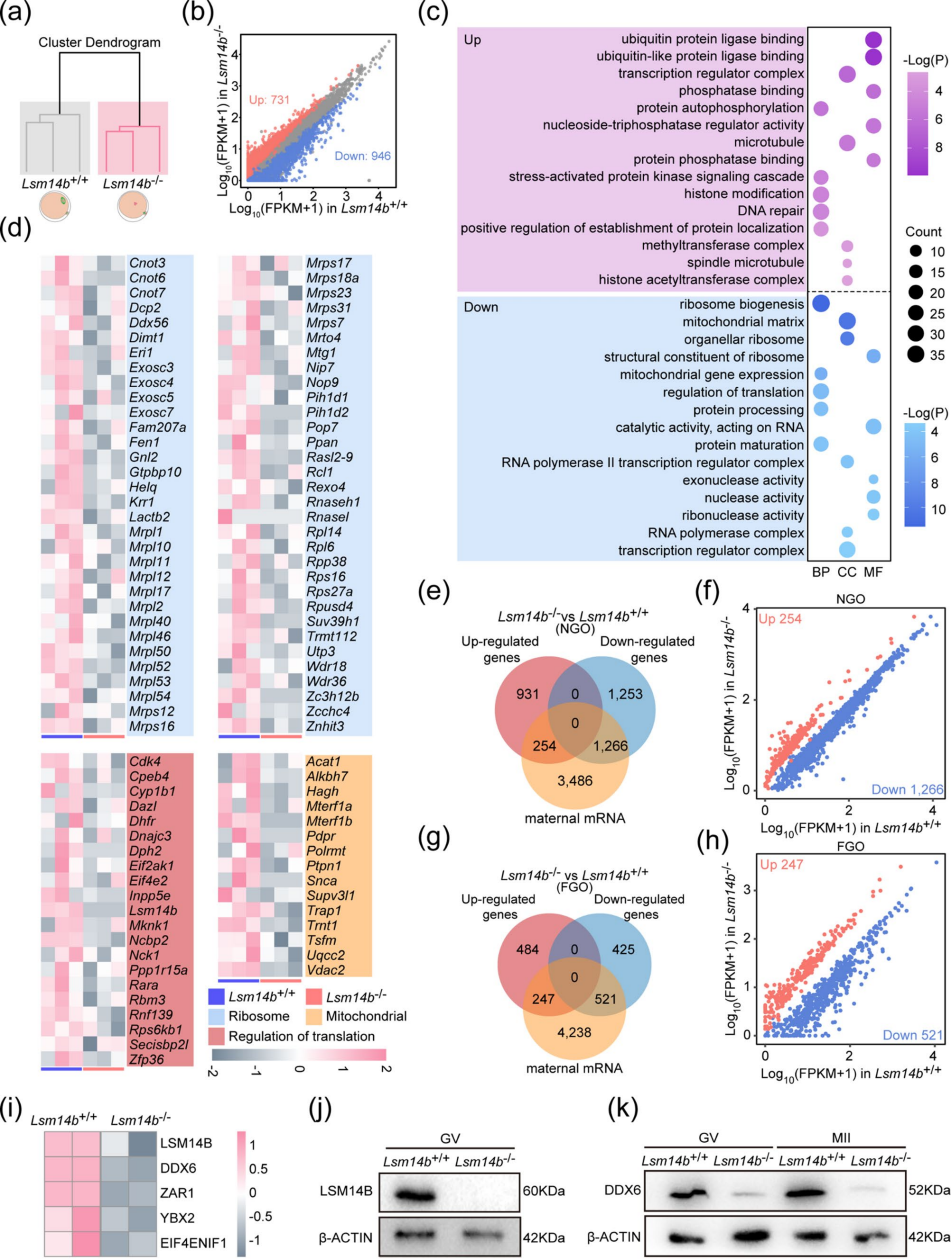
**LSM14B deletion impaired the maternal mRNA accumulation and translation in GV oocyte. a** Sample clustering of the transcriptome of RNA-seq data from GV oocytes. Gray color indicates WT oocytes, and pink color indicates *Lsm14b* KO oocytes. **b** Scatter plot comparing transcripts between WT and *Lsm14b* KO from GV oocytes. Transcripts decreased or increased in the *Lsm14b* KO oocytes, highlighted in blue or red, respectively. **c** Gene Ontology enrichment analysis of differential transcripts. **d** The heatmap shows the expression levels of “Ribosome,” “Mitochondrial,” and “Regulation of translational” related transcripts in WT and *Lsm14b* KO oocytes. **e** The Venn plot showing the overlap of maternal mRNA with differential genes of WT and *Lsm14b* KO NGO, where differential transcripts were acquired by scRNA-seq. **f** Scatterplot of transcripts between maternal transcripts in up-regulated and down-regulated in Fig. 5E. Down-regulated is displayed in blue; up-regulated is displayed in red. **g** The Venn plot showing the overlap of maternal mRNA with differential genes of WT and *Lsm14b* KO FGO, where differential transcripts were acquired by RNA-seq. **h** Scatterplot of transcripts between maternal mRNA in up-regulated and down-regulated in Fig. 5G. Down-regulated is displayed in blue; up-regulated is displayed in red. **i** The heatmap shows the expression of MARDO-associated proteins in WT and *Lsm14b* KO GV oocytes. **j** Detection of LSM14B and β-ACTIN proteins of GV oocytes by Western blot. **k** Detection of DDX6 and β-ACTIN proteins of GV and MII oocytes by Western blot

We performed RNA-seq analysis of GV-stage oocytes of *Lsm14b* KO and WT mice (Fig. 5a). A total of 1,677 transcripts were identified to be significantly changed, of which 946 transcripts were down-regulated and 731 were up-regulated, respectively, in the *Lsm14b* KO oocytes as compared with WT oocytes (Fig. 5b). By performing Gene Ontology analysis on the list of differential transcripts, we found that the expression levels of transcripts involved in “mitochondrial” and “translational functions” were significantly down-regulated. In addition, the expression levels of transcripts associated with protein phosphorylation were abnormal (Fig. 5c, d). Phosphorylation is a well-known mechanism for regulating the assembly and disassembly of membraneless compartments [39]. The Gene Set Enrichment Analysis (GSEA) results showed that in addition to most down-regulation of ribosome and translation-related transcripts, abnormal expression of transcripts involved in oocyte arrest, embryo development, and actin filaments could also be observed (Supplementary Fig. 5a, b). These results suggest that transcriptome was disrupted in *Lsm14b* KO oocytes. To further confirm whether the loss of *Lsm14b* affects the accumulation of maternal mRNA, we compared the differential transcripts of the two groups of NGO and FGO with the maternal mRNA, respectively. More maternal mRNAs could be observed to be down-regulated in *Lsm14b* KO NGO and FGO (Fig. 5e–h). The number of genes in the down-regulated maternal mRNA was nearly fivefold the number of up-regulated maternal mRNA genes (Fig. 5e, f) and is nearly twofold the number of up-regulated maternal mRNA genes in FGO (Fig. 5g, h), which also suggest that the structure of storing maternal mRNA in NGO and FGO is destroyed. A series of lines of evidence have shown that loss of *Lsm14b* affects the maternal mRNA accumulation and storage.

Considering that translation-related transcripts were significantly altered in FGO, we detected the protein level of GV oocytes. In protein mass spectrometry analysis of GV oocytes, we found significant clustering in WT and *Lsm14b* KO groups (Supplementary Fig. 5c). This further suggested that loss of *Lsm14b* affected translation in GV oocytes. Next, we performed GSEA on the differential proteins, and the results showed that mRNA metabolism, translation, and cell cycle-related proteins were significantly down-regulated (Supplementary Fig. 5d–e). These lines of evidence demonstrate that loss of LSM14B disrupts translational progression in GV oocytes. Furthermore, we observed that loss of *Lsm14b* resulted in down-regulation of protein levels of MARDO-associated proteins (EIF4ENIF1, YBX2, ZAR1, DDX6, and LSM14B) (Fig. 5i). It was also observed that the depletion of LSM14B leads to a decrease in DDX6 protein levels by Western blot detection of GV and MII oocytes (Fig. 5j, k). Collectively, these results suggest that the absence of LSM14B affects MARDO function and disrupts maternal mRNA accumulation and translation in GV oocytes.

### Defective meiotic progression in *Lsm14b* KO oocytes

**Fig. 6:**
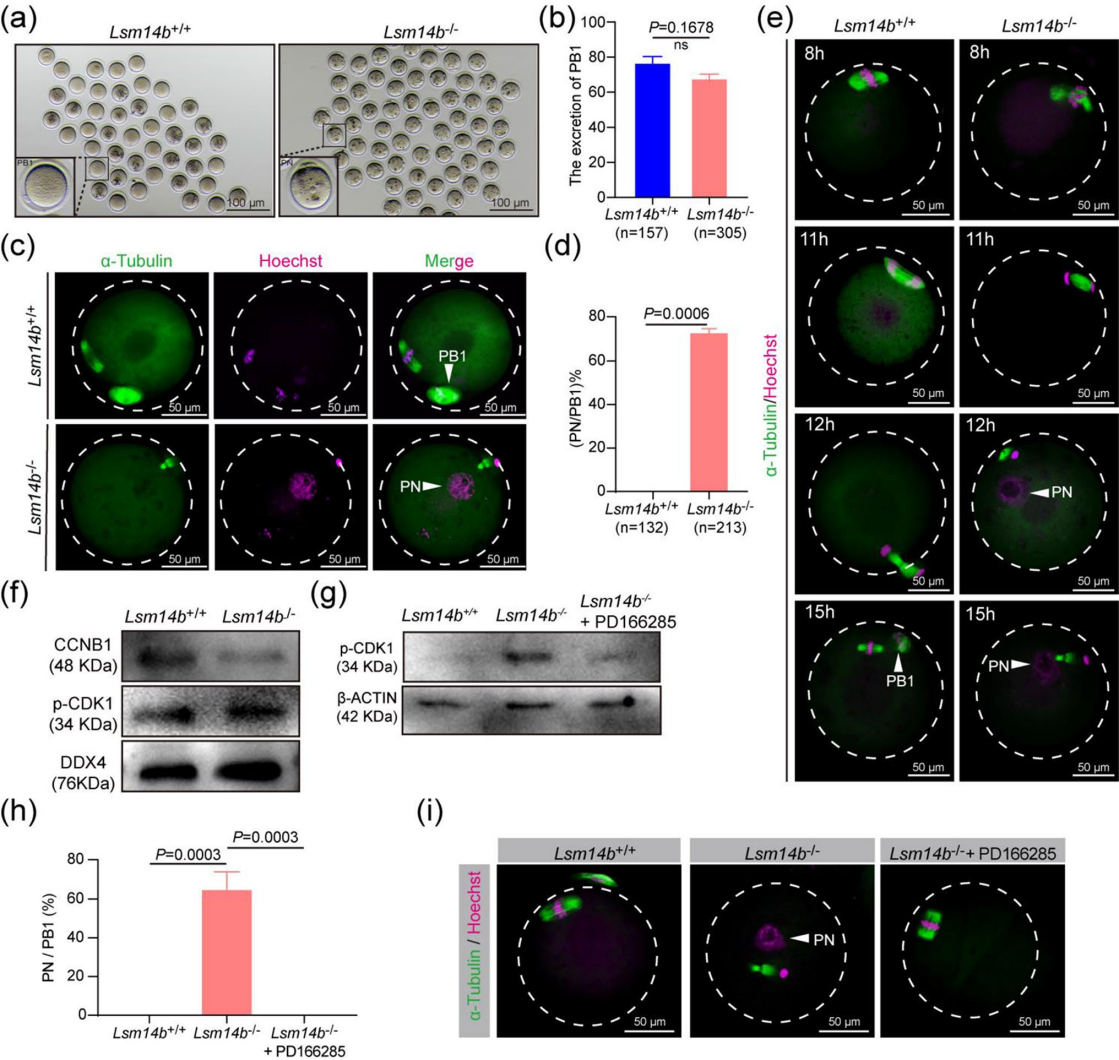
**Knockout of LSM14B interferes with oocyte meiotic progression. a** Representative image of WT and *Lsm14b* KO oocytes showed PB1 emission 15 h after hCG injection. Scale bar, 100 μm. **b** Percentage of PB1 released in WT and *Lsm14b* KO oocytes in *vivo*. Data are the mean ± SEM (*n* = 157 and 305 in the WT and *Lsm14b* KO oocytes, respectively). ns: non-significant. **c** Confocal microscopy results of WT and *Lsm14b* KO ovulated oocytes. Arrows indicate PB1 or PN. Spindle is labeled with α-Tubulin (green). Chromosome is labeled with Hoechst (magenta). Scale bar, 50 μm. **d** Percentage of PN/PB1 in WT and *Lsm14b* KO ovulated oocytes. Data are the mean ± SEM (n = 132 and 213 in the WT and *Lsm14b* KO oocytes, respectively). ns: non-significant. **e** Immunofluorescence showing spindle and chromosome in WT and *Lsm14b* KO oocytes after injecting hCG in different times. Scale bar, 50 μm. **f** Western blot results showing the levels of CCNB1 and p-CDK1 between WT and *Lsm14b* KO oocytes at MII stages. 200 oocytes are loaded in each lane. **g** Western blot results showed the levels of p-CDK1 and β-ACTIN in WT and *Lsm14b* KO oocytes by treatment with WEE kinase inhibitor, PD16685. 180 oocytes are loaded in each lane. **h** Percentage of PN/PB1 in WT, *Lsm14b* KO, and *Lsm14b* KO + PD166285 oocytes. (*n* = 3 biologically independent repeats). **i** Confocal microscopy results showing PN formation in WT, *Lsm14b* KO, and *Lsm14b* KO + PD166285 oocytes. Scale bar, 50 μm

*Lsm14b* KO female mice were found to be completely infertile (Fig. 2b), but the ovulation of *Lsm14b* KO female mice was normal (Supplementary Fig. 2 g). Given that oocyte quality is a key limiting factor in female fertility, the female infertility in *Lsm14b* KO female mice could be attributed to decline in oocyte quality [40]. Notably, we found that although *Lsm14b* KO oocytes completed meiosis I with normal cytokinesis and extruded morphologically normal PB1 in vivo almost of the *Lsm14b* KO oocytes contained a visible PN (Fig. 6a, b). IF staining revealed that residual central spindle was still visible between the decondensed chromatin and PB1 in nearly 80% of *Lsm14b* KO oocytes (Fig. 6c, d). It is well known that the spindle of the oocyte moves to the cortex and homologous chromosomes begin to separate during AI, and cytoplasmic division occurs when TI is reached; then meiosis enters MII with no intermediate phase [41, 42]. However, we found that oocytes fail to assemble the MII spindle after extrusion of the PB1 and enter into interphase and form a PN by using IF staining of oocytes after 8–15 h of hCG treatment (Fig. 6e). To confirm this result, GV oocytes were collected from ovaries and then cultured further in M16 medium for IVM. Consistent with the results in vivo, *Lsm14b* KO oocytes specifically form PN after cultured 16 h in vitro (Supplementary Fig. 6a, b).

Considering the MPFs (regulatory subunit CCNB1 and catalytic subunit CDK1) play indispensable roles in control of oocyte meiotic progression [43], we investigated the possibility whether knockout of *Lsm14b* affects the activation of MPF. We measured the levels of CCNB1 and p-CDK1 (Y15) in ovulated oocytes by Western blot analysis and found that the expression of CCNB1 was reduced and the levels of p-CDK1 (Y15) were significantly increased compared with WTs (Fig. 6f). Our results suggest that significantly increased levels of p-CDK1 (Y15) are probably the causes of the meiotic defects in *Lsm14b* KO oocytes. Previous studies have shown that PD166285, a specific inhibitor of WEE1/2 kinases, could be able to activate MPF by reduced levels of CDK1 (Y15) phosphorylation in mouse oocytes [44, 45]. Western blot analysis showed that PD166285 treatment dramatically reduced the levels of CDK1 (Y15) phosphorylation in *Lsm14b* KO GV oocytes at the end of 20 h IVM (Fig. 6g). Coincidently, treatment with the 10 µM PD166285 could prevent PN formation by the end of 20 h culture (Fig. 6h, i). These results confirmed that LSM14B is required for oocyte MII arrest.

## Discussion

The maternal mRNA is transcribed and transcripts accumulate during oocyte growth in mammals [46], and these mRNAs must be stored in membraneless compartments and then activated at the proper time in development. The role of membraneless compartments have been reported in non-mammalian oocytes, but less so in mammalian oocytes [8–11, 47, 48]. Maternal mRNA storage compartments have been characterized in non-growing and growing mouse oocytes and named this compartments the P-body-like granules [13]. In addition, it was previously reported that fully grown human and mouse oocytes accumulate mRNAs in a MARDO [34]. In this study, we identified that LSM14B is essential component of P-body-like granules and MARDO in mouse oocytes and required for the primordial follicle assembly and meiotic progression. Furthermore, we revealed that loss of LSM14B causes mRNA reduction in GO and disrupts maternal mRNA accumulation and translation in FGO. We propose that LSM14B is an important maternal factor and drives the maternal mRNA storage and stabilization during mouse oocyte growth.

In humans and mice, RNA transcription stops toward the end of oocyte growth and resumes after zygotic genome activation, during which stored maternal RNAs are used to make new proteins [49, 50]. Maternal mRNAs are thought to be stored in phase-separated membraneless compartments during oocyte growth and their stable storage is essential for successful meiosis maturation and early embryonic development [36, 51]. To date, membraneless RNA storage compartments have been partial characterized, and examples of such compartments include P granules and polar granules in non-mammalian oocytes [9, 47], as well as P-body-like granules and MARDO in mammalian early oocytes and FGO, respectively [13, 34]. Nevertheless, the function of the core component of the membraneless compartment has still not been fully dissected. We found that LSM14B co-localizes with DDX6 to P-body-like granules in germ cell. Recent studies demonstrated that the integrity of P-body-like granules is required for assembly of primordial follicles [13]. Consistent with this, the absence of *Lsm14b* leads to the dissolution and disappearance of P-body-like granules, consequently impacting the primordial follicles formation. Our results revealed that more than 50% of transcripts were found to be down-regulated in *Lsm14b* KO NGO. These data indicate that many mRNA are stored in the P-body-like granules and that disruption of P-body-like granules causes mRNA reduction. Interestingly, the down-regulated mRNAs in *Lsm14b* KO germ cells were associated with processes related to “mitochondrion organization” and “oxidative phosphorylation.” It is demonstrated that depletion of ZAR1 disrupts MARDO formation and mitochondrial clustering [34]. Given that LSM14B serves as a core component of the MARDO, further study should investigate the potential effects of LSM14B deficiency on the morphology and distribution of mitochondria in mammalian oocytes.

Oocyte meiotic maturation and early embryonic development rely on maternal mRNAs stored in FGO [52]. Previous studies have shown that loss of YBX2 in mouse oocytes leads to developmental retardation and failure of normal maturation of oocytes [35]. Notably, YBX2 is a RNA-binding protein, and YBX2 deficiency mainly reduces mRNA stability, thereby disrupting maternal mRNA accumulation [35]. The other study confirmed that ZAR1/2 are required for oocyte meiotic maturation by regulating the maternal transcriptome [6]. Our study showed that loss of LSM14B caused a large number of dysregulated maternal mRNA and meiotic defects. In addition, some of the aberrant transcripts in *Lsm14b* KO FGO were associated with “cell cycle arrest” and “embryo development,” However, LSM14B acting as an RNA-binding protein to bind which maternal mRNA needs to be explored in mammalian oocytes. It is demonstrated that the deficiency of ZAR1/2 in FGO impairs the 3’-UTR activation of maternal mRNA, thereby affecting the translation of maternal mRNA and causing oocyte meiotic defects [6]. In addition, previous study confirmed that maternal mRNAs stored in the MARDO are translationally repressed [34]. They also found that tdPCP-LSM14B also represses translation. In the present study, transcripts associated with ribosomes and translation are suppressed in *Lsm14b* KO FGO. Protein mass spectrometry analysis of FGO revealed that deletion of LSM14B alters the oocyte proteome. Collectively, these data indicate that LSM14B is essential for translation in mammalian oocyte.

Low levels of MPF activity are one of the causes of PN formation and meiotic defects [45]. Our observation that the *Lsm14b* KO oocytes failed to assemble the MII spindle after extrusion the PB1, but instead entered the interphase and formed PN. Furthermore, WB analysis of the p-CDK1 revealed that the levels of p-CDK1 are significantly reduced in *Lsm14b* KO MII oocytes. Likewise, *Eml1* knockdown (KD) impairs MPF activity around the time of oocyte resumption, ultimately leading to the formation of PN [45]. Similarly, *Mastl* is required for the rapid rise of CDK1 activity that is needed for the entry into MII stage, and *Mastl*-null oocytes reassembled a nuclear structure containing decondensed chromatin and failed to enter MII because they maintain high levels of p-CDK1 [44]. It is therefore rational to believe that the absence of LSM14B destroyed the activity of MPF at optimal levels responsible for PN formation. This inference is further buttressed by the observation that PD166285, a WEE1/2 kinases inhibitor, effectively prevented the CDK1 (Y15) phosphorylation and the formation of PN in the *Lsm14b* KO oocytes. It is not clear how LSM14B may regulate the activity of MPF in oocytes. It could be that loss of LSM14B disrupts maternal mRNA stability and expression in GV oocyte. Nevertheless, the role of LSM14B during oocyte meiotic maturation is unclear. Whether LSM14B is essential for timely maternal mRNA degradation remains to be explored in mammalian oocytes.

## Supplementary Information

Below is the link to the electronic supplementary material.

Supplementary file1 (DOCX 28 KB)

## Data Availability

The FGO proteome and P7 ovaries Co-IP-MS data have been deposited to the integrated proteome resources (iProX) and the dataset identifiers PXD040510 and PXD040513, respectively. The scRNA-seq and RNA-seq data have been deposited in the Genome Sequence Archive under accession CRA009935 and CRA010004, respectively. All data needed to evaluate the conclusions in this work are present in the main text or the supplementary materials. The R code using for data processing is publicly available at GitHub at https://github.com/ZZlab412/Lsm14b-KO-mice-sequencing-data-analysis-script.
