# Supplementary material for: LSM14B controls oocyte mRNA storage and stability to ensure female fertility
Source: Cell Mol Life Sci. 2023 Aug 14;80(9):247. doi: 10.1007/s00018-023-04898-2 (PMC10425512; doi:10.1007/s00018-023-04898-2)
Supplement: Supplementary file 1 — Supplementary file1 (DOCX 28 KB) [file 18_2023_4898_MOESM1_ESM.docx]

**Supplemental Figures**

**Fig. S1 Expression analysis of membraneless compartment associated proteins.**

**a** The table shows the scores of membraneless compartment associated proteins bound to LSM14B using Co-IP followed by mass spectrometry analysis. **b** The line chart of P-body components expressed with developmental timeline (E14.5, E16.5, E18.5, P1, P3, P4, P6).

**Fig. S2 LSM14B expression and knockout phenotype analysis.**

**a** Quantitative RT-PCR results showing the relative expression levels of *Lsm14b* in oocytes and other somatic tissues. Data are the mean ± SEM (n = 4 biologically independent repeats). **b** Gene-targeting strategy for CRISPR/Cas9-based mouse *Lsm14b* knockout. F, forward; R, reverse. **c** Representative image of WT and *Lsm14b* KO mice at 6-week-old. **d** The PCR products amplified from genomic DNA extracted from mouse tail samples were analyzed. M, marker. **e** The line chart of body weight with developmental timeline (2 weeks, 3 weeks, 6 weeks, 8 weeks) in the WT and *Lsm14b* KO mice. Data are the mean ± SEM (n = 3 females for each genotype). **f** Representative image of the ovaries of WT mice and *Lsm14b* KO mice at 8-week-old. **g** Quantitative analysis of ovulation number of WT mice and *Lsm14b* KO mice at 8-week-old. Data are the mean ± SEM (n = 3 females for each genotype). ns: non-significant. **h** Representative H&E image of different classes of follicles in 6-month-old ovaries. Scale bar, 100 μm.

**Fig. S3 Ovarian single cell sequencing information and quality control results.**

**a** The sequenced detail information of 2 samples after CellRanger and Seurat workflow. **b** Violin plot demonstrating the number of genes (nFeature_RNA), unique molecular identifier (nCount_RNA), and percentage of mitochondrial genes (percent.mt) in the different data sets. **c** The distribution of ovarian cells in the two groups (left). Clustering of ovarian cell population with UMAP, colored based on 14 cell clusters (right). **d** The bubble chart shows the expression of genes specifically expressed in different cell clusters. The size of the dot represents the percentage of cells expressing the specified gene in each cluster, and the color intensity of the dot represents the average expression level of the specified gene. **e** Cluster analysis of germ cells with UMAP plots based on transcriptional patterns. **f-g** Expression of *Id1* and *Ooep* in each set with germ cell branches.

**Fig. S4 Changes in transcription profiles of *Lsm14b* KO granulosa cell.**

**a** Cluster analysis of granulosa cells with UMAP plots based on transcriptional patterns. **b** UMAP map of WT and *Lsm14b* KO granulosa cell subpopulations. **c** Single-cell pseudotime developmental trajectory of WT and *Lsm14b* KO granulosa cells, which are colored according to cell development state. **d** Volcano plots of gene differential expression at granulosa cell between pairs of WT vs. *Lsm14b* KO. **e** The Gene Ontology enrichment of DEGs in granulosa cell. **f** The KEGG enrichment of DEGs in granulosa cell.

**Fig. S5 Transcriptome and protein mass spectrometry analysis.**

**a** GSEA results for differential transcripts. **b** Scatterplot of the 6 terms (highlighted in red) in A). Down-regulated is displayed in blue, up-regulated is displayed in red, respectively. **c** PCA of GV oocyte shotgun mass spectrometry. Red color indicates WT oocytes, and blue color indicates *Lsm14b* KO oocytes. **d** GSEA results of differential proteins. **e** Heatmap shown protein expression of key terms in WT and *Lsm14b* KO GV oocytes.

**Fig. S6** **Phenotype of *Lsm14b* KO oocytes *in* *vitro* maturation**

**a** Immunofluorescence showing spindle assembly and chromosome arrangement of WT and *Lsm14b* KO oocytes at 16 h culture. Arrows represent PB1 or PN. Spindle is labeled with α-Tubulin (green), Chromosome is labeled with Hoechst (magenta). Scale bar, 50 μm. **b** Percentage of PN/PB1 in WT and *Lsm14b* KO oocytes in *vitro*. Data are the mean ± SEM (n=132 and 138 in the WT and *Lsm14b* KO oocytes, respectively)

**Table S1. Primers used for PCR analyses**

The primer sequence is to identify the mouse genotype and to detect the mRNA level of *Lsm14b.*

**Table S2. Antibody information**

Information on antibodies used in the research.

**Table S1. Primers used for PCR analyses**

| **Gene** | **Forward primer sequence (5-3’)** | **Type of application** |
| --- | --- | --- |
| *Lsm14b*-F1 | CTATTAACACCTGGGCCAGAAGG | genotyping |
| *Lsm14b*-R1 | AAGTAGCAGTATGAGGGTTTCCAG | genotyping |
| *Lsm14b*-F2 | ATCGTGACCCTTAGAATACCACAG | genotyping |
| *Lsm14b*-F | GAAGTTGGTAAGCCCTCCAGCCTCA | RT-qPCR |
| *Lsm14b-*R | GTGAGCTGAGGTGGCTCTGAGGACA | RT-qPCR |
| *Gapdh*-F | GCTACACTGAGGACCAGGTTGTCT | RT-qPCR |
| *Gapdh*-R | GAGGTCCACCACCCTGTTGC | RT-qPCR |

The primer sequence is to identify the mouse genotype and to detect the mRNA level of *Lsm14b.*

**Table S2. Antibody information**

| **Antibody** | **Code** | **Company** |
| --- | --- | --- |
| LSM14B | NBP2-76362 | Novous |
| DDX4 | ab27591 | Abcam |
| DDX6 | 14632-1-AP | Proteintech |
| p-CDK1-Y15 | AP0016 | Abclonal |
| α-Tubulin-FITC | F2168 | Sigma |
| NOBOX | sc-514178 | Santa |
| LHX8 | ab137036 | Abcam |
| IgG | 2729 | CST |
| CCNB1 | AB72 | Abcam |
| β-Actin | 4970 | CST |
| GAPDH | 60004-1-IG | Proteintech |
| HRP-conjugated goat anti-Mouse | 31430 | Thermofisher |
| HRP-conjugated goat anti-Rabbit | 31460 | Thermofisher |

Information on antibodies used in the research.
